# Determinants of antiretroviral therapy adherence among transgender women in South Africa

**DOI:** 10.4102/safp.v66i1.5869

**Published:** 2024-04-29

**Authors:** Leonashia Leigh-Ann van der Merwe, Idah Moyo, Azwihangwisi H. Mavhandu-Mudzusi

**Affiliations:** 1 Department of Health Studies, College of Human Sciences, University of South Africa, Pretoria, South Africa; 2 Department of HIV Services, Population Solutions for Health, Harare, Zimbabwe; 3 School of Social Sciences, College of Human Sciences, University of South Africa, Pretoria, South Africa

**Keywords:** ART adherence, determinants, HIV, continuity in treatment, transgender women

## Abstract

**Background:**

Transgender women bear a huge burden of human immunodeficiency virus (HIV) in South Africa. However, they are not fully engaged in healthcare across the HIV continuum of care. In addition, transgender women face multiple facets of stigma and discrimination as well as socio-economic inequalities, which all have a negative impact on antiretroviral therapy (ART) adherence.

**Objective:**

The study aimed at exploring and describing the experiences of ART adherence of transgender women living with HIV in the Buffalo City Metro Municipality.

**Methods:**

The study employed an interpretative phenomenological analysis (IPA) design. Twelve participants were enrolled using a snowballing sampling technique. Data were collected using semi-structured interviews and analysed using an IPA framework.

**Results:**

While exploring determinants to ART adherence among transgender women living with HIV in Buffalo City Metro, two superordinate themes emerged: enablers to ART adherence and psychosocial factors promoting adherence. The study found that factors such as differentiated ART service delivery, ARV medicines-related factors, motivators for taking treatment and support systems facilitated ART adherence.

**Conclusion:**

Emerging from this study is the need to scale up differentiated, person-centred ART service deliveries that will enhance access and adherence to treatment for transgender women.

**Contribution:**

This study provides unique insights on factors enhancing ART adherence among transgender women. There is a paucity of literature on access to HIV care services for key and vulnerable populations, and these findings will be shared in the country and in the region.

## Introduction

The Joint United Nations Programme on HIV/AIDS (UNAIDS)^1^ stipulates that in 2022 there were 1.3 million new human immunodeficiency virus (HIV) infections worldwide, adding up to a total of 39 million people living with HIV (PLWH). The UNAIDS^1^ reported that, in 2022, East and Southern Africa remains the region most heavily affected by HIV accounting for more than half (54%) of PLWH globally. According to the UNAIDS,^1^ South Africa has the largest HIV epidemic in the world. In 2021 in South Africa, an estimated 7.5 million people (all ages) were living with HIV and the country had an HIV prevalence rate of 18.3%. According to the UNAIDS,^1^ in 2022 the HIV prevalence for transgender persons in South Africa was 58%.

The World Health Organization^2^ highlights the significance of early initiation on antiretroviral therapy (ART), treatment continuity, ART adherence and HIV viral suppression in improving the quality of life for HIV-positive individuals. Therefore, HIV treatment is critical in reducing the spread of HIV and contributing to the attainment of the control of the HIV epidemic.^2^ Despite these initiatives to end AIDS by 2030, studies in Indonesia^3^ and in Zimbabwe^4^ have demonstrated that key populations experience barriers in accessing HIV services, including ART initiation and low ART adherence.

The term *transgender* is an umbrella term that refers to a person whose gender identity or expression differs from that assigned by society at birth.^5^ Scheibe et al.^6^ define transgender women as individuals who were assigned male at birth but socially identify as women. They further argue that gender is a social construct powerful enough to drive HIV infection.^6^ According to Jimmy and Jose^7^ medication, adherence refers to the degree to which a person’s behaviours comply with the recommendations of a healthcare practitioner.

A South African study^8^ estimated the HIV prevalence among transgender women to be 52% in 2019. The Buffalo City Metro Municipality accounts for 46% HIV prevalence among transgender women.^9^ Although this group has a higher HIV rate, transgender women have less access to HIV care and are also less likely to be retained in HIV care.^10^ Despite a disproportionate burden of HIV infection, studies reveal that there is low uptake of HIV testing among transgender women.^11,12^ Another related study^11^ established that transgender people avoid healthcare facilities because they perceive these spaces as being unsafe. A cross-sectional survey conducted in the United States found comparatively lowest healthcare utilisation among transgender women and that gender-affirming treatments were sought outside of the traditional, supervised medical setting.^13^ An analysis of data^6^ for the HIV outreach programme targeting transgender women in Buffalo City Metro Municipality shows a high ART defaulter rate among them. There is paucity of literature that describes ART adherence among transgender women.^14^ To facilitate treatment continuity and retention in care for the transgender women on ART, the researchers found it essential to explore and describe determinants of ART adherence among transgender women living with HIV in Buffalo City, South Africa, exploring all the steps across the prevention and treatment cascade in this community.

## Research methods design

An interpretative phenomenological analysis (IPA).^15,16^ facilitated an in-depth exploration of narratives capturing the lived experiences of transgender women as they navigated the healthcare system to access HIV care. The IPA approach has a threefold focus: interpretive, double hermeneutic and idiographic nature.^16^ It also focuses on examining the lived experience of the individual by drawing from the concepts of phenomenology, hermeneutics and idiography.^17^ These three aspects enable the researcher to explore and understand the phenomenon under study and consider the unique experience of each participant.^16^ The IPA enabled the researchers to have an in-depth understanding of individual transgender women’s experiences in accessing HIV care services.

### Study setting and participants

This study participants were HIV-positive individuals who self-identified themselves as transgender women accessing HIV care in the Buffalo City Metro Municipality, Eastern Cape province, South Africa. Of the 12 study participants, three were on hormonal therapy prescribed from the study setting. The Buffalo City was selected as it was one of the data-collection sites for the first ever bio-behavioural survey to determine HIV prevalence among transgender women in South Africa. The Eastern Cape is one of the poorest provinces in South Africa, although it is endowed with rich mineral resources, high agricultural production and a world-renowned manufacturing base.^18^ The estimate is that in 2018, the HIV prevalence for the Buffalo City Metro Municipality was 14.46%,^19^ in contrast to the 46% HIV prevalence among transgender women in the Buffalo City Metro Municipality. The demographic data of the participants are displayed in Table 1.

**TABLE 1:** The demographic data of study participants.

Participant	Thuli	Se	Lucy	Liz	Lee	Roe	Aya	Pee	Joy	Pink	Busi	Thae
Marital status	Single	Single	Single	Single	Single	Single	Single	Married	Single	Single	Single	Single
Occupation	Peer Educator	Unemployed	No work	-	Student	Self employed	Temp work	Case Manager	Sex worker	Unemployed	Unemployed	Peer Educator
Age	30	32	24	28	23	29	31	31	31	42	25	31
Race	Black	Black	Black	Black	Black	Coloured	African	African	Black	Black	Black	Black
Sex at birth	Male	Male	Male	Male	Male	Male	Male	Male	Male	Male	Male	Male
Current identity	Trans	Transgender	Female	Trans	Transgender	Gay/Trans	Transgender	Transgender woman	Transgender woman	TG	Gay/Trans	Queer
Religion	Zion	Christian	Christian	Christian	Christian	Christian	Christian	Christian	Christian	Christian	Christian	-
Number of sexual partners	2	6	1	2	1	3	1	6	>50	1	3/4	1
Sex of intimate partner	Male	-	Male	Male	Male	Male	Male	Gay man	Male	Male	Male	Male
Highest level of education	Grade 8	Grade 10	Grade 11	Grade 10	Grade 12	Grade 10	Tertiary Education	N6 Public Management	Grade 11	Grade 11	Grade 12	Grade 12
Romantic or sexual relationship?	Romantic	Sexual	Romantic	Romantic	Romantic	Sexual	-	Both	Both	Romantic	Romantic	Sexual
Please estimate your monthly income	R5940	R800	No income	R2000	R350	R1000	R1500	R10 600	R3000	N/A	R300 or less	R6000
When did you learn of your HIV positive status?	2003	2006	2020	2013	2019	2019	2015	2010	2007	2004	2019	2008
Are you on antiretroviral medication?	Yes	Yes	Yes	No	Yes	Yes	Yes	Yes	Yes	Yes	Yes	Yes
How long have you been on antiretroviral meds?	2 years	2 years	3 months	-	1 year	2 years	5 years	10 years	13 years	16 years	3 months	5 years
Have you had any gender affirming procedures such as hormones and/or surgery?	None	None	None	Hormone treatment	No	No	No	None	Hormone treatment	Hormone treatment	No	No
If yes, how long have you been taking gender affirming hormones?	N/A	N/A	N/A	3 years	N/A	N/A	N/A	N/A	-	-	N/A	N/A

### Data collection

An interview guide facilitated data collection. The development of the interview guide was based on literature review and the study purpose.^16^ The interview guide questionnaire consisted of semi-structured, open-ended questions. Initially, a pilot study was conducted, involving two transgender women who were not included in the study. Through this process, the researchers were able to refine the interview guide. Data were collected between March 2020 and June 2020. Each interview lasted for approximately 60 min and was conducted in English. However, participants were allowed to express themselves in their mother tongues, which were mainly Xhosa and Afrikaans where they had some challenges as the interviewer is fluent in both languages. Fortunately, all of them expressed themselves in English that eliminated the issue of translation. The interviews took place at the researcher’s office to ensure privacy and to enable adherence to social distancing in accordance with coronavirus disease 2019 (COVID-19) protocols. The interviews were audio recorded with the consent of study participants.

The interviews were constructed around one request: What were *your experiences of using* ART services as a transgender woman living with HIV? In order to elicit more detailed descriptions on the phenomenon under study, the interview guide had probing questions such as ‘What is your experience with ARVs?’ and, ‘Can you tell me how that experience made you feel?’ and ‘How do you cope?’ ‘What support system did you have?’. Interviews were conducted until data saturation was reached at the ninth participant, that is the point at which no new analytical information comes forth and maximum information on the phenomenon is provided by the participants.^20^ Three additional participants were interviewed to establish whether there would be any new information. A total of 12 transgender women participated in the study.

### Data analysis

The data were transcribed verbatim into written text. Interpretative phenomenological analysis enabled detailed exploration of how HIV-positive transgender women made sense of their HIV care experiences. The focus of IPA is to examine the lived experiences of the individual by drawing from the concepts of phenomenology, hermeneutics and idiography. This approach has been utilised in related studies and proved to be useful in exploring participants’ experiences.^16^ Each transcript was analysed independently by three researchers (L.L.-A.v.d.M., I.M. and A.H.M-M.), using the IPA framework as described by Smith and Osborn.^16^ An outsider, but academic expert on IPA, was requested to act as an independent co-coder and given audio recordings and transcripts to compare. Each researcher read the transcript several times and listened to the audio recording a few times. The following steps, outlined by Smith and Osborn,^16^ were followed: (1) reading and re-reading the transcript, (2) note-taking and developing emergent themes, (3) clustering the emergent themes, (4) crafting a master table of themes composed of superordinate themes, subthemes and extracts from the interviews (5) examining and comparing divergent and convergent themes, constructs and ideas from the master tables of the themes and (6) compiling a single master list composed of a superordinate theme, themes, and subthemes. Following this process, the independent coder was given a table of themes from all researchers to work on. The research team then met to compare and discuss the preliminary list of superordinate and subordinate themes and developed a detailed description of their meanings. The team had a consensus and agreed on the final master table, composed of superordinate themes, themes and subthemes and related excerpts from the transcripts (Table 2). This was to ensure credibility.^21^

**TABLE 2:** Superordinate themes, themes and subthemes.

Superordinate themes	Themes	Sub-themes
Enablers to ART adherence	Differentiated ART service delivery	Treatment delivered at home Use of central chronic medication dispensing and distribution
	Medicines-related factors	Frequency of taking treatment Absence of side effects Knowledge of treatment
Psychosocial factors promoting adherence	Motivators of taking treatment	Fear of death Desire to live Media messages Disclosure of HIV status
	Support systems	Partner support Support From family members who are living with HIV

ART, antiretroviral therapy.

### Trustworthiness

Measures were taken to ensure trustworthiness and rigour as enunciated by Maher et al.^22^ To ensure trustworthiness, the research should satisfy four criteria, namely credibility, transferability, dependability and confirmability.^22^ According to Shosha,^23^ credibility refers to the accuracy of the study findings. All interviews were audio recorded and transcribed verbatim to enhance credibility. In addition, member checking, peer evaluation and use of an independent co-coder further enhanced credibility as advocated for by Lincoln et al.^24^

Beck^25^ indicates that transferability is the ability of the findings to be transferred to other contexts. This was achieved by the provision of detailed descriptions of the research setting and context. To enhance dependability, thick descriptions were provided, and safe keeping of research documents ensured an audit trail that can facilitates replication of the study by any other researcher. Confirmability was enhanced by the use of bracketing to minimise researcher bias.

It is important to recognise that the experiences of participants are understood through the subjective interpretation of the researcher.^23^ The first author who collected data is experienced in qualitative research. The researcher involved a lot of introspection and internal examination to explore personal feelings, experiences and biases and all these were bracketed to enhance confirmability. This approach allows one to become less assuming about another’s experience, to be open, compassionate and non-judgemental and present data from the perspectives of the study participant, thus ensuring credibility.^21^ Moreover, as the study was part of a Masters dissertation, the supervisor guided all the process through guiding the formulation of an interview guide, listening to the first two pilot interviews and provided guidance regarding proper questioning and probing to avoid channelling the participants to respond in a particular manner. In addition, peer debriefing was carried out following every interview, and irregularities identified were addressed. An independent co-coder experienced in qualitative research (who was outside the context of the study) listened to the audio-recorded interviews. She went on to review and assess the transcripts to check for the emerging and final categories from those transcripts and the final themes. Consensus was reached on the final master table of themes. This afforded the interviewers the opportunity for continued introspection throughout the process. By coding and recoding many times, comparing the themes and categories with the co-coder, the researchers ensured dependability. To enhance authenticity, verbatim excerpts from the interviews were used to substantiate themes emanating from the transcripts. Throughout, all the steps of analysis, interpretations and ideas were discussed by the research team and the independent co-coder to ensure that the process was as robust as possible. In addition, the research team also met to discuss and compare the initial findings and agreed on the super and subordinate themes.

### Ethical considerations

Protecting the rights of the institution is an important principle of research.^23,24^ To ensure that this principle was achieved, the researcher sought and obtained ethical clearance from the University of South Africa Africa’s College of Human Sciences Research Ethics REC-012714-039 (NHERC). Permission to conduct the study was also obtained from the Health and Social, Health and Empowerment Feminist Collective of Transgender Women based in the Buffalo City Metro Municipality. As part of the consent process, participants were informed that participation was voluntary and that the interviews would be audio recorded. Participants were also informed that they were free to decline or discontinue participation at any time, if they wished to do so. Participants were assured that data would be handled confidentially and that the results would be reported in a way that ensured anonymity. Therefore, all identifiers were removed from the transcripts and pseudonyms used. Data were stored in a password-protected computer. The study participants were provided with a detailed information sheet that explained the details of the study. They then gave an informed written consent after reading and understanding all the information.

## Findings

Two superordinate themes emerged from data analysis: enablers to ART adherence *and* psychosocial factors promoting adherence.

### Enablers to antiretroviral therapy adherence

#### Factors promoting adherence

This superordinate is about factors that acted as enablers in ART adherence. The superordinate comprised two themes: differentiated ART service delivery and medicines-related factors.

**Differentiated antiretroviral therapy service delivery:** This theme focuses on differentiated service delivery approaches that promote adherence to ART. These factors include treatment delivered at home and use of Central Chronic Medication Dispensing and Distribution. Having treatment delivered at home makes it easier for transgender women to access and be adherent to HIV treatment.

***Treatment delivered at home:*** Decentralised ART is a delivery approach where treatment is dispensed to the client outside of a health facility; an approach useful to reach key populations, who might otherwise not be engaged in care.^26^ The use of peers to reach transgender clients was preferred by participants, as illustrated in the text:

‘The work done by NGOs; they call us closer to our return dates and reminding us to constantly take our treatment accurately, and to maintain good nutrition is what make sure that I am taking treatment.’ (Thuli)

‘There were some peers that worked with the NGOs [non-*governmental organisation*] and participated in the delivery of ARV medicines in the community. This became a convenient way of getting the medicines close by in the community.’ (Liz)

Other participants found it easier to collect medication at a central point outside of the health facility, with reduced waiting times, like a drop-in centre.

***Use of central chronic medication dispensing and distribution:*** The central chronic medication dispensing and distribution programme is a differentiated service delivery model in which clinically stable HIV-positive patients can access their medications through a service provider outside of a health facility.^26^ This approach was formulated in response to an overburdened healthcare system and to reduce waiting times in the context of treatment dispensing. The programme is useful for transgender women who do not have to interface with healthcare workers very often to collect their treatment, as stated by this research participant:

‘CCMDD is easy, it is an easy way of getting the medication. So, that’s how they are making people take their treatment. It is an easier way than those long queues at the clinic. Because at the clinic someone is sick with flu, the other one with TB, the other one with many, many diseases. So, you are there just to take your medication then go, but then you will be staying there. You are at risk of contracting the diseases of other patients at the clinic.’ (Se)

**Medicines-related factors:** This theme is about antiretroviral therapy (ARVs) medicines-related factors that contributed to treatment adherence. It has three subthemes: frequency of taking treatment, absence of side effects and knowledge of treatment.

***Frequency of taking treatment:*** In this study, frequency of treatment refers to the rate at which medication is taken, as per prescription of a health provider. A participant revealed that the new ART regimen facilitated more consistent doses, resulting in improved adherence, as captured in the text:

‘I feel very great about these new ones, especially the ones you take in the morning. We don’t take them in the evening anymore. They are called TLD. Before the current ones, we had to take them in the evening, because they would make you feel drunk. With the current ones, you can take them in the morning and still go to work without experiencing any side effects. It is like you have taken a headache tablet.’ (Lee)

Transgender women living with HIV reported the absence of side effects to be an enabler of ART adherence.

***Absence of side effects:*** The presence of these side effects is a powerful barrier to ART adherence;^9^ in the same way that the absence of these effects is a powerful facilitator of ART adherence. In this study, it is illustrated by the quote:

‘I’ve never experienced side effects because when they first gave me my medication, they told me that sometimes you will get side effects. You will hallucinate, it’s been how many years now since 2015 and I’ve never had any issues.’ (Aya)

Having knowledge of treatment also facilitates adherence to treatment.

***Knowledge of treatment:*** Knowledge is a critical component to ensure treatment literacy among transgender women.^27^ Treatment literacy is defined as the degree to which individuals have the ability to find, understand and use information related to major issues associated with an illness or disease, such as the science, treatment, side-effects and guidelines. This makes patients more responsible for their own health.^28^ A study^28^ found that treatment literacy not only denotes the capacity to take medication effectively but also to prevent HIV infection, as well as HIV-related stigma and discrimination. In this study, participants revealed their knowledge of treatment to be an enabling factor for ART adherence:

‘What is making me stick to my treatment currently is that I know that if you do not take your treatment the virus will multiply in your body. Also, if you take treatment, then stop it, you are opening an opportunity for your body to be drug resistant, and in that time the virus will not have stopped, it will be continuing to spread. That is what motivate me to take my treatment.’ (Pink)

**Motivators for taking treatment:** This theme is about factors that act as motivators for adhering to treatment. It has four subthemes: fear of death, desire to live, media messages and disclosure of HIV status.

The fear of morbidity and mortality prompted transgender women to be compliant with taking treatment.

***Fear of death:*** Getting sick and dying is a powerful motivator for ART adherence among PLWH.^29^ The same is true for transgender women, as quoted:

‘I know I’m doing good because it’s my life. Throwing the tablets under the bed can kill me, so if I want to live a good life, then I have to take my treatment. Putting medication under the bed, did kill some of the people. I don’t want to be like those people.’ (Busi)

In addition to multiple enablers, transgender women expressed the desire to live as motivation for adhering to medication.

***Desire to live:*** The desire to live and be healthy facilitates ART adherence.^30^ The participants in this study were also motivated to adhere to ART in order to live a happy and fulfilling life:

‘I’m not going to live a life for somebody else because this is my life. When I take those medicines, I’m taking them to remain well and to be healthy. So, I am not going to put my life in somebody else’s hands. That’s not going to help.’ (Pee)

The exposure to HIV media messaging plays an important role in communicating the importance of adhering to HIV treatment.

***Media messages:*** The media plays a critical role in the fight against HIV/AIDS. In particular, radio messaging not only serves as a memory aid to those taking ART but can also be powerful in communicating the importance of ART adherence.^31^ The use of radio messages is a facilitator of ART adherence, as stated by this participant:

‘What motivated me was listening to the radio and PHY. PHY was once at NU1 hall. I was one of the speakers on stage, with one of my friends Bianca, we were talking about defaulting, HIV positive people, transgender women, everything. So, they are the ones that motivated me actually, because it was said that if you default, when you have to take the medication again, it will not work. I then got scared. I got scared since then, I never defaulted again.’ (Pink)

Some participants felt that living openly with HIV is an important factor for medication adherence.

***Disclosure of human immunodeficiency virus status:*** Living openly with HIV or ‘positive living’ is a colloquial term used by persons living with HIV, referring to situations where people reveal their HIV status openly without stigma and/or shame. Positive role modelling has been a key strategy in providing peer-to-peer services among transgender women who live with HIV, as a way of reaching their peers to increase retention in care.^6^ Many PLWH fear stigma, societal stigma and discrimination, but living openly with HIV could be a source of strength, as articulated in the quote:

‘I want to do what I was telling others to do, because I used to tell people to use treatment. So, why must I not practise what I used to preach? So that’s how I take my treatment, I take it with a smile. Oh! I love it, I love it.’ (Roe)

**Support system:** Two subthemes emerged: partner support and support from family members living with HIV.

***Partner support:*** This subtheme refers to any kind of support received from romantic and/or sexual partners of transgender women. Partners play a very particular support role in terms of adherence to ART.^27^ The participants revealed that the role of a partner motivated them to remain adherent to treatment:

‘No, my boyfriend knows my everything. He knows everything about me. He knows that I am taking ARVs, and I am not hiding anything from him. He also reminds me to take my medication.’ (Joy)

Having the support of family members who are living with HIV was vocalised as an enabling factor for ART adherence among transgender women living with HIV.

***Support from family members who are living with human immunodeficiency virus:*** Treatment support can manifest itself in the form of peer support from family members or friends who are also living with HIV.^30^ Study participants revealed that treatment adherence support came from family who are themselves HIV-positive, as depicted further in the text:

‘So, there was this cousin of mine in the house, who was taking ARVs. He was giving me a lot of support and was my pillar of strength so, going to the clinic, taking his treatment for him. He would send me on some other days to go and take his ARVs.’ (Thae)

## Discussion

Our findings document the diverse individual, social, and structural factors influencing HIV treatment adherence among transgender women in South Africa. To our knowledge, this is one of the few studies that has qualitatively examined adherence and treatment continuity among this vulnerable group in Southern Africa. It contributes to the growing body of literature in Southern African countries about a group that carries one of the highest HIV burdens among key populations.^3^ It also seeks to better understand the determinants of ART adherence among transgender women engaged in HIV care services.^5,32^

This section discusses the results in relation to the determinants of ART adherence among transgender women living with HIV. The participants reported many factors that promote adherence to ART. Participants verbalised that the frequency of ART doses motivated their adherence to medication. This is contrary to studies elsewhere, where transgender women are found to be less adherent to HIV medication.^33,34^ A sentiment shared by participants in this study is that familial, intimate partner and broader social support can lead to improved adherence to ART and improved health outcomes. These findings are similar to those of an Indonesian study by Fauk et al.^35^ that established that social support from transgender peers and family members played a crucial role in enhancing ART adherence among transgender women. Participants cited treatment knowledge as an enabler for adherence to ART, a finding corroborated by a study of Jamaican transgender women.^32^ The desire to live, fear of death and the physical impact of HIV were found to be salient factors in promoting ART adherence, confirmed by a Guatemalan study.^36^ Participants also reported the absence of treatment side effects as an enabler of medication adherence. Treatment side effects can lead PLWH, including transgender women,^9^ to be less adherent to treatment. Participants also indicated that they preferred the new regimen (dolutegravir [DTG]-based) because of less side effects. The World Health Organization^37^ recommends the use of DTG as the preferred ART regimen for naïve individuals because of its efficacy and safety profile, reduced adverse events and improved adherence and treatment continuity.

Participants reported decentralised medication distribution as a motivating factor for adherence to treatment. The Central Chronic Medicine Dispensation and Distribution programme employs a decentralised approach in which clients who are clinically stable (determined virologically and meeting appointments) can access ART at a distribution point of their choice.^24,38^ Related to this, Macdonald et al.^26^ recommend the use of such innovative differentiated service delivery approaches to enhance HIV services and improve ART adherence among key populations. Participants reported satisfaction with this approach as they do not have to go to the health facility to collect ART. This limits possible contact with healthcare workers and minimises potential stigma and discrimination from healthcare workers and fellow patients alike. Consistent with a review that demonstrated that memory aids such as phones and radios are positively associated with improved adherence,^30^ participants reported that radio messaging acted as a motivator in taking treatment and thus facilitated adherence to ART. In addition, there is a very strong argument on the role of healthcare providers in packaging messages on medication adherence through printed media (e.g., flyers, posters), social networking sites (e.g., Facebook, Twitter and Instagram), text messages, mobile applications (e.g., WhatsApp and Telegram), televisions and radios.^39^

## Conclusion

This is one of the first qualitative studies exploring the determinants of adherence to ART for transgender women in South Africa. It emerged from this study that differentiated, person-centred ART service delivery is a crucial component in enhancing access and adherence to treatment for transgender women. The insights from this study talk to the need for policymakers, programme planners, and providers of HIV care to be conscious of the medicine related and psychosocial factors that promote ART adherence among transgender women. The research findings highlight the importance of involving transgender women in developing specific HIV programmes for them.

### Limitations of the study

It is important to acknowledge the study limitations. The study was limited only to transgender women who accessed ART in Buffalo City South Africa; therefore, the findings refer to the experiences in this city. While the existing literature on transgender persons and access to HIV care is limited, that which is available points to the fact that the experience of transgender persons in South Africa and in the region is similar.^6,8,12^

### Implications of the study

The implications are that enabling factors such as differentiated service delivery approaches, medicine related and psychosocial determinants are critical in developing strategies that enhance treatment adherence and continuity. Key to the study findings is that service provision for whom the program is being crafted must be underpinned by an understanding of the communities to enhance the provision of differentiated person-centred HIV care.
